# Molecular dynamics simulations involving different β-propeller mutations reported in Swiss and French patients correlate with their disease phenotypes

**DOI:** 10.1038/s41598-024-75070-4

**Published:** 2024-10-15

**Authors:** Finola Priyadharshini Chandrasekaran, Everette Jacob Remington Nelson

**Affiliations:** Gene Therapy Laboratory, Department of Integrative Biology, School of Bio Sciences and Technology, Vellore Institute of Technology, Vellore, 632 014 India

**Keywords:** αIIbβ3, Β-propeller, Missense mutations, Molecular docking, Molecular dynamics, Glanzmann Thrombasthenia, Biophysics, Computational biology and bioinformatics, Structural biology, Diseases, Medical research

## Abstract

Integrin αIIbβ3 is the predominant receptor for fibrinogen which mediates platelet aggregation, an important step in hemostasis and thrombosis. Several mutations have been reported in the genes encoding αIIb and β3 subunits among patients with Glanzmann thrombasthenia, of which 177 are in the β-propeller domain. The two subunits form a heterodimer at the interface between β-propeller and β-I domains of αIIb and β3, respectively with their stability critical for intracellular trafficking, surface expression, and ligand binding. Our study was aimed at retrieving the β-propeller mutations from various databases and studying structural variations due to select mutations upon interaction with fibrinogen using molecular docking and molecular dynamics. Mutations were studied for their impact on phenotypic severity, structural stability, and evolutionary conservation. Molecular docking analysis and molecular dynamics simulations were carried out for αIIb-β3 complexes as well as αIIbβ3-fibrinogen complexes; in particular, E355K structure had more deviations, fluctuations, and other changes which compromised its structural stability and binding affinity when compared to both wild-type and G401C structures. Our comprehensive in silico analysis clearly reiterates that mutations in the β-propeller are not only responsible for structural changes in this domain but also have implications on the overall structure and function of integrin αIIbβ3.

**Supplementary Information:**

The online version contains supplementary material available at 10.1038/s41598-024-75070-4.

## Introduction

**Figure 1:**
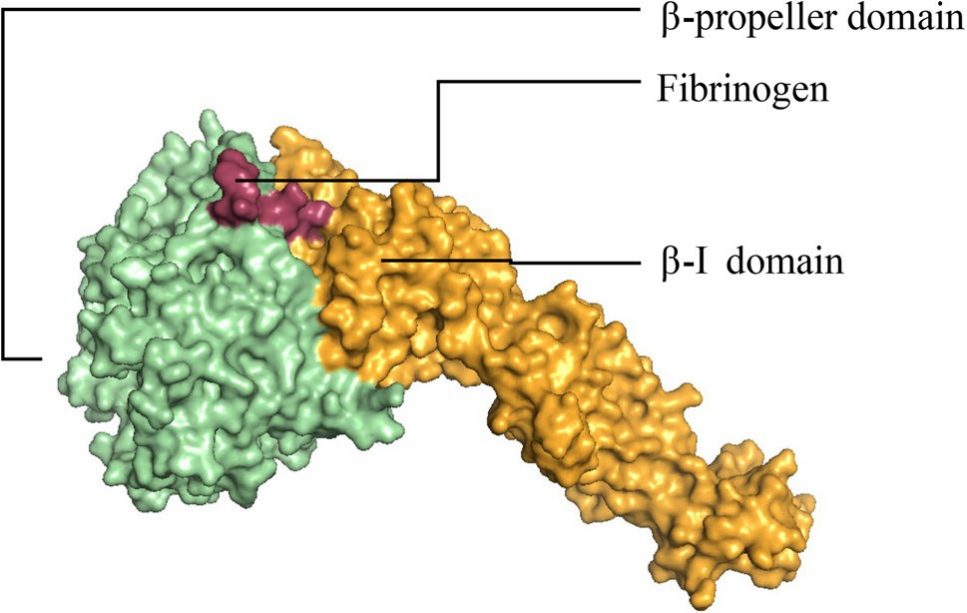
Depiction of fibrinogen (γC peptide) interacting domains of αIIbβ3^4^.

Absent or abnormal surface expression or ligand binding of integrin αIIbβ3 results in an inherited bleeding disorder called Glanzmann thrombasthenia (GT). Hemostasis which refers to a physiological mechanism that arrests bleeding following injury to a blood vessel occurs in two phases: primary and secondary. Primary hemostasis as well as pathologic thrombosis (blood clot formation) is initiated by small, enucleated blood cells called platelets. Platelets recruited to the site of a ruptured blood vessel undergo adhesion (to vessel wall) and aggregation (to each other). Integrin αIIbβ3 is the predominant receptor for mediating platelet aggregation transitioning from a low-to-high affinity state for binding with fibrinogen and other ligands in response to soluble agonists (inside-out signaling). Ligand binding triggers integrin clustering leading to platelet spreading, irreversible aggregation, and clot retraction (outside-in signaling). Fibrinogen binding regions are localized to the N-terminal portions of integrin αIIbβ3. The upper face of the ectodomain constituting the headpiece of integrin αIIbβ3 has β-propeller and β-I domains that contain ligand binding sites (Fig. 1)^1^. The β-propeller maintains stability of the protein structure of αIIbβ3 which is attributed to β-sheets in its secondary structure. The β-propeller structure consists of seven blades arising from consecutive amino acid repeats each of which includes 4 antiparallel strands with Ca^2+^ ions in every blade^2^. While blades 1–3 are essential for associating with the β-I domain of β3, blades 4–7 do not seem to have much importance albeit the presence of Ca^2+^ ions. In addition, the β-propeller has seven FG-GAP repeats of about 60 amino acids harboring putative Ca^2+^ binding motifs^3^.

The β-propeller domain is important for biogenesis, defects in which have often been associated with GT types 1 and 2. N15-linked glycosylation in the β-propeller was reported to control pro-αIIb maturation, heterodimerization, and degradation^5^. The N15-glycated pro-αIIb heterodimerizes with β3 in the endoplasmic reticulum (ER) which is followed by carbohydrate modification and proteolytic cleavage in the Golgi apparatus to form mature αIIbβ3 complexes^6^. Mutations in the β-propeller were found to abrogate complex formation thereby preventing their transport from the ER to Golgi, which implies the intricate role of this domain^7–12^. Structural modifications caused by the mutations G128S, S287L, and G357S that variably impaired progression of pro-αIIbβ3 complexes from the ER to Golgi were characterized in our previous study^13^.

Integrin αIIbβ3 is capable of binding to ligands, such as fibrinogen, fibrin, von Willebrand factor, and fibronectin. Fibrinogen is a physiologically important ligand that is involved in crosslinking activated platelets, a vital step in hemostasis. The binding event happens between the γ-C peptide in fibrinogen and interface of the “cap” region containing 4 loops on the top side of the αIIb β-propeller domain and “specificity determining loop” in the β3 β-I domain^14^. The ligand binding sites require Ca^2+^ and Mg^2+^ ions for proper binding of αIIb and β3 subunits. Most of the Ca^2+^ ions are located on the blades of β-propeller determining the stability and folding of the structure. The residues 1-334 of the β-propeller were reported to regulate fibrinogen binding. Earlier, peptide crosslinking residues 171–464 and 294–314 in recombinant αIIb highlighted the importance of these residues in ligand binding. Most of the naturally occurring mutations involving the residues 145–224 of the β-propeller affected ligand binding^15^.

In particular, mutations in the residues 184–193 of the third amino terminal repeat blocked fibrinogen binding^16^. Among them, D163A was found to affect ligand binding by causing a defect in folding, while T176I, Y143H, and P145A^4,16^ also led to a failure in binding with fibrinogen. Most of the GT mutations reported so far are associated with the β-propeller domain, which shows its indispensable contribution to the structure and function of the integrin αIIbβ3. The aim of this study was to retrieve missense mutations in this domain from various databases and highlight structural changes due to select mutations upon interaction with fibrinogen using molecular docking and molecular dynamics.

## Results

### Mutation screening

A total of 177 single nucleotide polymorphisms (SNPs) obtained from the various databases were located within amino acid residues 1-451 of the αIIb subunit corresponding to the β-propeller domain (Table S1).

### Pathogenicity analysis

All identified SNPs were further analyzed for their pathogenicity using PredictSNP^17^, out of which sixty were predicted to be deleterious and pathogenic using tools, such as PredictSNP, MAPP, PhD-SNP, Polyphen-1, Polyphen-2, SIFT, and SNAP (Table S2). Not surprisingly, most of these mutations that were associated with specific functional defects correlated with GT type 1.

### Stability analysis

Mutations affecting the structural stability of a protein are often responsible for the disease phenotype. Hence, mutations with deleterious and pathogenic consequences were studied further. These mutations were analyzed for their impact on structural stability using the iStable^18^ tool, which is used for analyzing the changes in stability as a result of the mutations. Twenty-seven mutations were found to destabilize the structure of integrin αIIbβ3 based on the DDG scores obtained using tools, like MuPro, iStable and I-Mutant (Table S3).

### Conservation analysis

**Figure 2:**
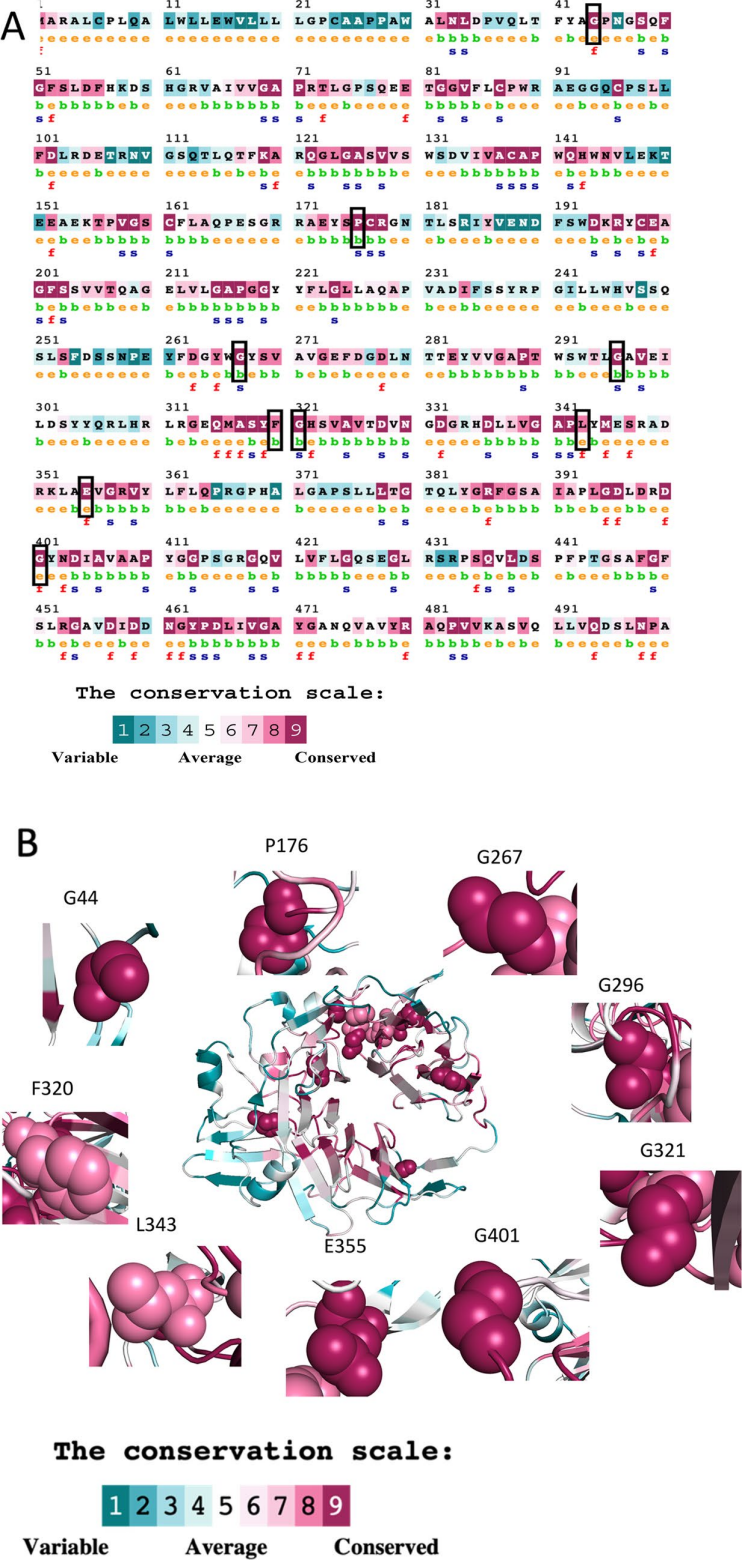
Evolutionary conservation analysis of the αIIb amino acid sequence (**A**) with the highly conserved amino acid residues depicted on the β-propeller structure (**B**).

Amino acid residues that are highly conserved during the course of evolution are unlikely to be mutated. Accordingly, conservation analysis was done for all the identified mutations and only those that involved highly conserved residues were studied further. Nine mutations had occurred in highly conserved regions as indicated (Fig. 2A). Among these, while residues G44, E355, and G401 are highly conserved and exposed on the β-propeller surface, residues P176, G267, G296, and G321 although highly conserved, are buried. The other residues, namely F320 and L343 are also conserved and buried (Fig. 2B). Although these mutations were reported to be functionally important, structural changes caused by them have not been studied in detail.

### Molecular docking studies

**Figure 3:**
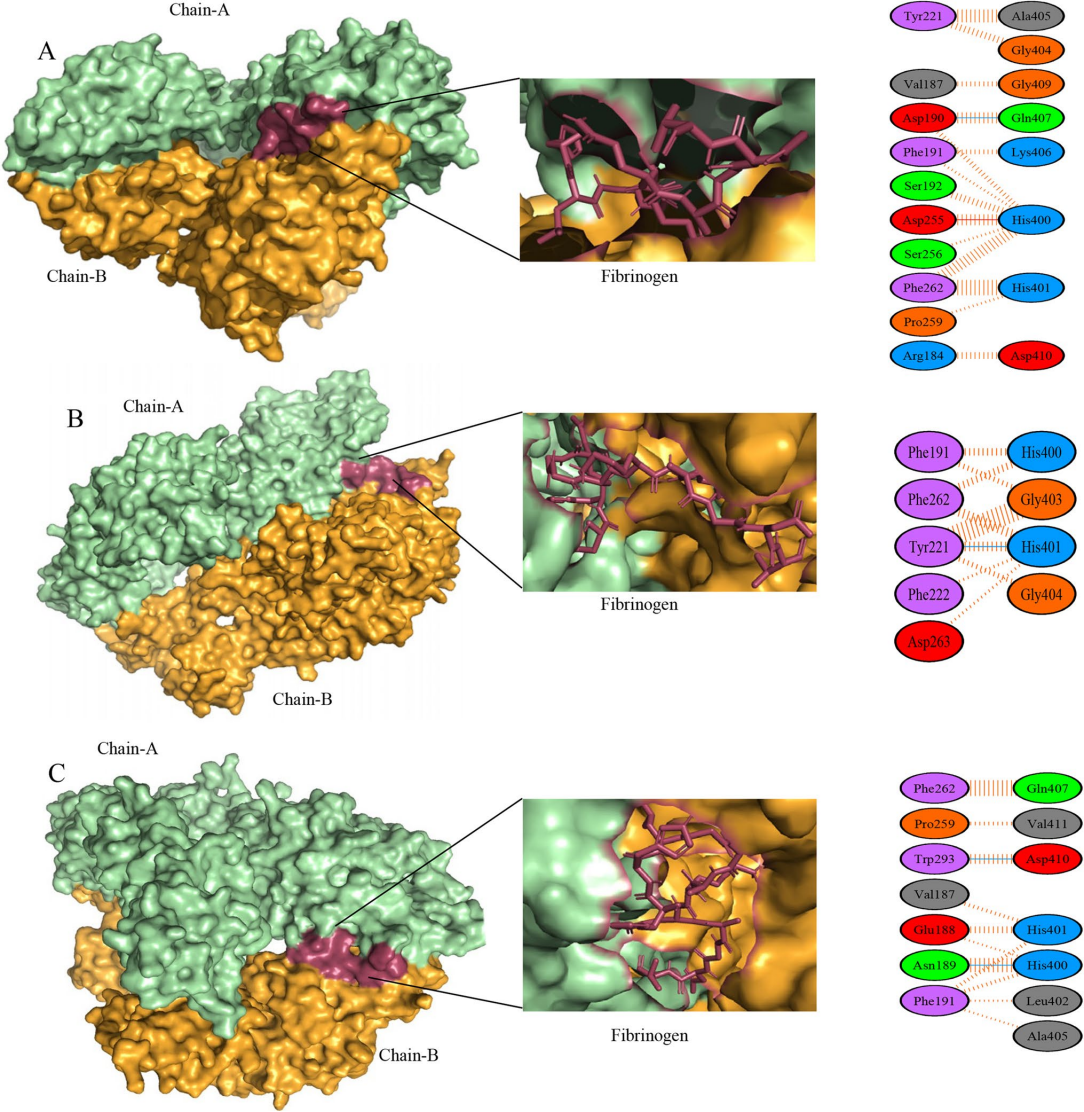
Three dimensional interactions of αIIbβ3 complexes after docking with fibrinogen. Wild-type (**A**), E355K (**B**), and G401C (**C**). Chains A and B represent aIIb and b3 subunits, respectively.

Some of the prominent and functionally significant mutations reported in the literature were chosen for this study (Table 1). The wild-type, G296R, F320S, G321W, E355K, and G401C αIIbβ3 structures were subjected to molecular docking analysis, which revealed the binding energy, docking score, and binding orientation between the αIIb and β3 subunits (Table 2). In general, mutant structures with lower binding energies were found to form highly stable complexes with greater binding affinity. Among the five mutations, E355K and G401C had the highest binding energies indicating that these two mutations might affect fibrinogen binding to mutant αIIbβ3 complexes when compared to the wild-type due to changes in the interactions (Fig. 3A-C). Therefore, E355K and G401C mutations were considered for MD simulations to better understand their deleterious consequences as detailed below.

**Table 1:** Prominent β-propeller mutations causing Glanzmann thrombasthenia.

Accession number	Mutation	I-Mutant	MUpro	I-Stable	PredictSNP	MAPP	PhD-SNP	PolyPhen-1	PolyPhen-2	SIFT	ConSurf	Refs.
CM061068	G296R	D	D	D	D	D	D	D	D	D	8	^19^
CM153672	F320S	D	D	D	D	D	D	D	D	D	9	^20^
CM030472	G321W	D	D	D	D	D	D	D	D	D	9	^34^
CM981073	E355K	D	D	D	D	D	D	D	D	D	9	^35^
CM153663	G401C	D	D	D	D	D	D	D	D	D	9	^34^

D – Deleterious / Destabilizing

**Table 2:** Docking scores of wild-type, G296R, F320S, G321W, E355K, and G401C αIIbβ3 structures.

Protein structure	HADDOCK score	Van der Waals energy	Electrostatic energy
Wild-type	-59.0 +/- 28.9	-66.0 +/- 9.8	-371.3 +/- 92.5
G296R	-163.4 +/- 4.4	-83.6 +/- 5.1	-391.8 +/- 28.1
F320S	-139.7 +/- 4.2	-100.3 +/- 16.6	-423.8 +/- 65.6
G321W	-177.3 +/- 35.2	-102.0 +/- 12.3	-569.8 +/- 91.5
E355K	-60.0 +/- 38.9	-76.0 +/- 10.8	-361.3 +/- 136.5
G401C	-124.3 +/- 42.5	-85.0 +/- 20.7	-687.0 +/- 101.4

### MD trajectory analysis

#### E355K compromised stability as compared to G401C

**Figure 4:**
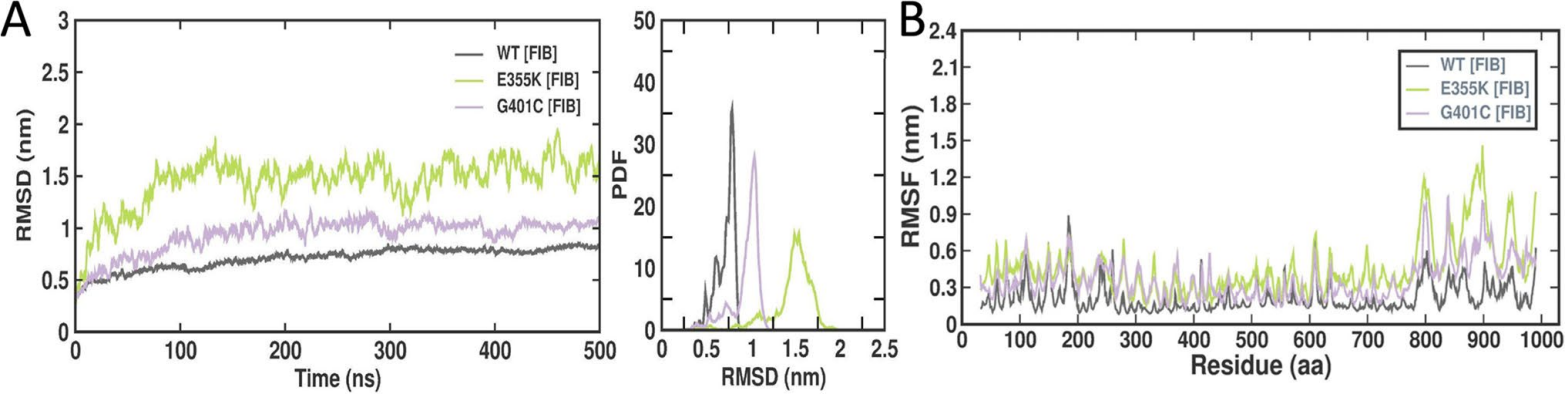
RMSD and RMSF plots of wild-type, E355K, and G401C αIIbβ3 structures bound to fibrinogen following MD simulations (500 ns). (**A**) RMSD values corresponding to the fibrinogen-bound wild-type, E355K, and G401C αIIbβ3 complexes. The x-axis represents time in ns, while the y-axis represents RMSD values in nm. (**B**) Graphical representation of RMSF values corresponding to the fibrinogen-bound wild-type, E355K, and G401C αIIbβ3 complexes. The x-axis indicates amino acid residues, while the y-axis indicates RMSF values in nm.

RMSD was calculated to determine the convergence and deviations with time based on Cα atoms in the wild-type, E355K, and G401C αIIbβ3 structures bound to fibrinogen (Fig. 4A). The average RMSD values of E355K and G401C αIIbβ3 complexes with fibrinogen were observed to be higher as compared to that of the wild-type (Table 3). The protein structures were stabilized with fixed values which indicated that the docked complexes of E355K and G401C had deviations throughout the simulation period (500 ns), with E355K recording the highest fluctuation. Similarly, RMSF was calculated which indicated the changes in flexibility of the wild-type, E355K, and G401C αIIbβ3 structures over time when bound with fibrinogen. The E355K structure displayed more flexibility implying stability changes when compared to the wild-type, with the G401C structure showing slightly less fluctuation (Fig. 4B). The average RMSF values of E355K and G401C αIIbβ3 complexes with fibrinogen were observed to be higher as compared to that of the wild-type (Table 3).

**Table 3:** Average RMSD, RMSF, Rg, and SASA values of fibrinogen-bound wild-type, E355K, and G401C αIIbβ3 complexes.

Protein structure	RMSD (nm)	RMSF (nm)	Rg (nm)	SASA (nm^2^)
Wild-type	0.71 +/- 0.10	0.22 +/- 0.11	3.79 +/- 0.04	673.33 +/- 29.10
E355K	1.45 +/- 0.25	0.45 +/- 0.22	4.25 +/- 0.09	746.03 +/- 15.69
G401C	0.94 +/- 0.16	0.36 +/- 0.16	4.11 +/- 0.08	718.12 +/- 16.48

**Figure 5:**
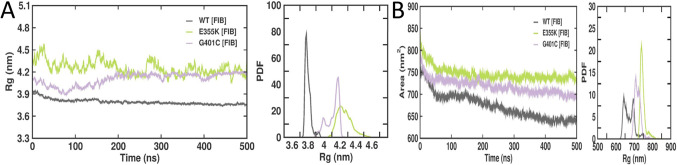
Rg and SASA plots of wild-type, E355K, and G401C αIIbβ3 structures bound to fibrinogen following MD simulations (500 ns). (**A**) Rg values corresponding to the fibrinogen-bound wild-type, E355K, and G401C αIIbβ3 complexes. The x-axis represents time in ns, while the y-axis represents Rg values in nm. (**B**) SASA values corresponding to the fibrinogen-bound wild-type, E355K, and G401C αIIbβ3 complexes. The x-axis indicates time in ns, while the y-axis indicates area in nm^2^.

The Rg value of a protein structure is used to calculate the distribution of atoms from the centre of the mass, which denotes the compactness of the protein structure. The competence and folding of the wild-type, E355K, and G401C αIIbβ3 structures bound to fibrinogen were observed at different time points during the trajectory, which revealed that when compared to the wild-type fibrinogen-bound αIIbβ3 complex, both E355K and G401C complexes exhibited more deviations thereby compromising the compactness of the protein structure (Fig. 5A). The average Rg values of fibrinogen-bound wild-type, E355K, and G401C αIIbβ3 complexes were 3.83, 4.31, and 4.11 nm, respectively (Table 3). SASA was performed in order to identify the changes caused by mutations in the hydrophobic core of the protein structure, which showed changes in SASA associated with both fibrinogen-bound E355K and G401C complexes as compared to the wild-type (Fig. 5B). The average SASA values of fibrinogen-bound wild-type, E355K, and G401C αIIbβ3 complexes were 673.33 +/- 29.10, 746.03 +/- 15.69 and 718.12 +/- 16.48, respectively (Table 3). An increase or decrease in the SASA values is indicative of an impact on the protein structure. Accordingly, the E355K mutation with a higher SASA value resulted in an enlarged solvent-accessible surface area in the E355K structure.

**Figure 6:**
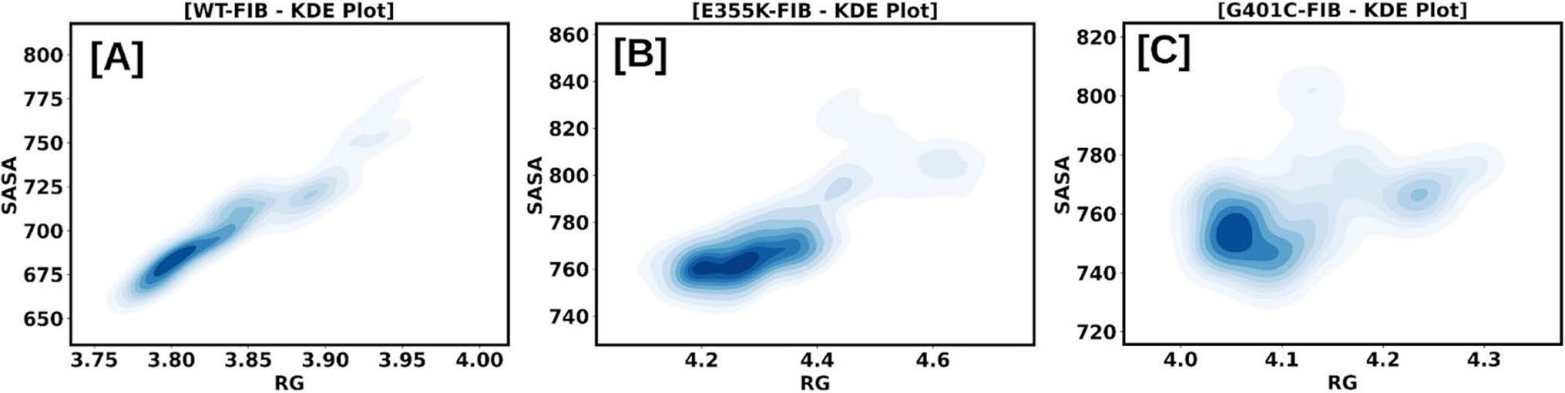
KDE plots of Rg and SASA are represented together as collective variables for fibrinogen-bound wild-type (**A**), E355K (**B**), and G401C (**C**) αIIbβ3 complexes.

Compactness and flexibility of the protein structure are reciprocally connected to each other. Rg and SASA were calculated to study the accessible surface area of mutant structures for the solvent. Kernel density estimation plots of Rg and SASA for wild-type, E355K, and G401C αIIbβ3 structures were plotted, which revealed significant differences in the mutant structures when compared to the wild-type (Fig. 6).

#### Intermolecular H-bond

**Figure 7:**
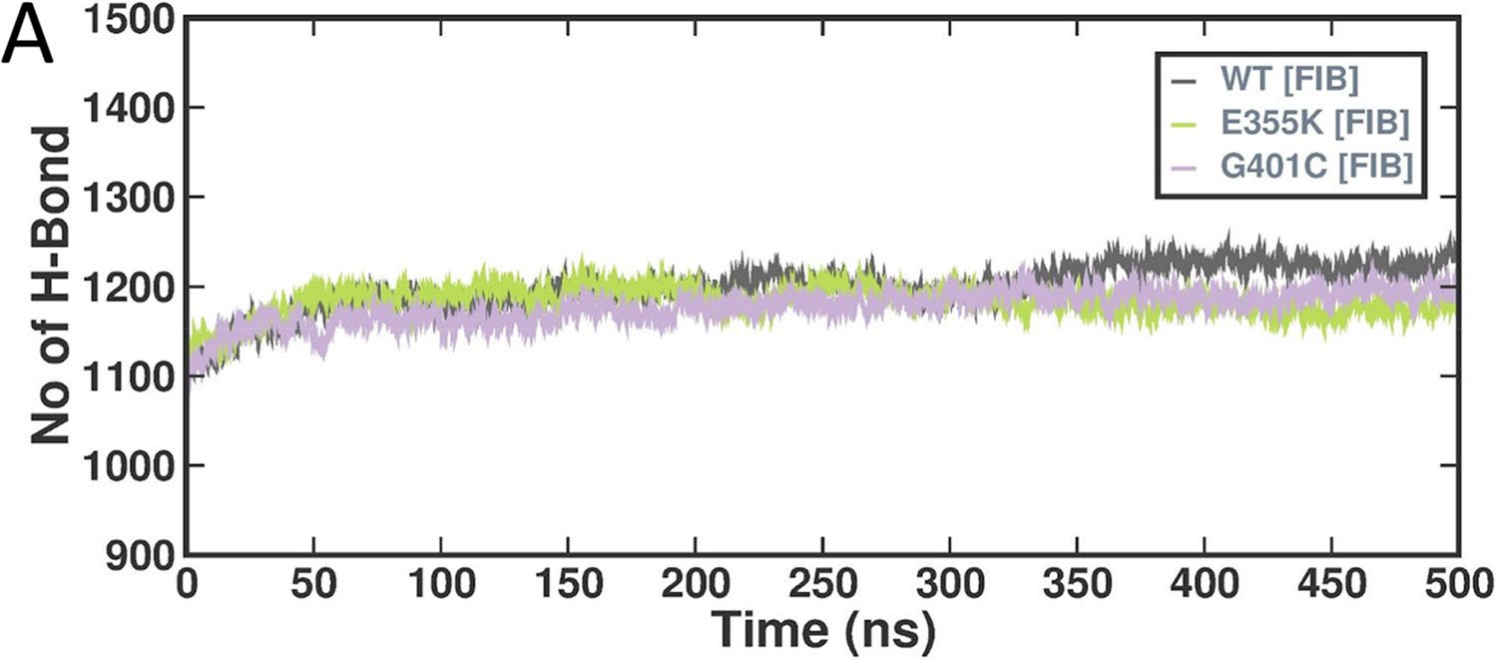
Graphical representations of H-bond interactions of wild-type, E355K, and G401C αIIbβ3 structures bound to fibrinogen following MD simulations (500 ns). The x-axis represents time in ns, while the y-axis represents number of H-bonds.

The hydrogen bonds present in a protein are responsible for maintaining its structure and determining its binding specificity. Intermolecular hydrogen bonds were observed with respect to the wild-type, E355K, and G401C αIIbβ3 structures bound to fibrinogen which indicated changes in interactions after 350 ns in the mutant complexes when compared to the wild-type (Fig. 7).

#### Secondary structure elements

Mutations causing changes to the secondary structure of the fibrinogen-bound wild-type and mutant αIIbβ3 complexes were evaluated. The structural behavior of a protein may be influenced by the proportion of secondary structure elements, like α-helixes, β-sheets, turns, etc. Overall, the E355K and G401C complexes had slightly more of these structures, mainly β-sheets, when compared to the wild-type (Table 4).

**Table 4:** Secondary structure elements in fibrinogen-bound wild-type, E355K, and G401C αIIbβ3 complexes.

Protein structure	Total	Coil	β-sheet	β-bridge	Bend	Turn	α-helix	3-helix
Wild-type	50	31	30	3	18	9	7	1
E355K	53	28	32	3	18	10	8	1
G401C	53	28	33	2	18	10	8	1

#### E355K affected fibrinogen binding

Binding free energy was calculated using the molecular mechanics Poisson-Boltzmann surface area (MM-PBSA) approach to study the energy association between the αIIbβ3 and fibrinogen structures throughout the MD simulation period. Although the average binding energies for the E355K and G401C structures were found to be lower when compared to the wild-type structure (Table 5A), binding energies associated with active residues involved in ligand binding were higher for these mutant structures when compared to that of the wild-type (Table 5B).

**Table 5:** A. Overall binding energies of fibrinogen-bound wild-type, E355K, and G401C αIIbβ3 complexes. B. Hotspot interaction binding energies of fibrinogen-bound wild-type, E355K, and G401C αIIbβ3 complexes.

**A**			
**Energy (kJ/mol)**	**Wild-type**	**E355K**	**G401C**
Van der Waals energy	-171.246 +/-10.092	-224.196 +/- 16.584	-230.061+/-9.585
Electrostatic energy	-0.145+/-5.530	-28.530+/-12.109	-26.105+/-7.410
Polar solvation energy	69.868+/-12.438	149.086 +/-35.490	138.083 +/-22.505
SASA energy	-18.322+/-0.852	-22.691 +/-1.174	-23.408+/-0.874
Total binding energy	-119.845+/-11.238	-126.331+/-22.628	-141.491+/-17.615
**B**			
**Energy (kJ/mol)**	**Wild-type**	**E355K**	**G401C**
Van der Waals energy	-121.87+/-38.925	-80.56+/-13.234	-84.43+/-12.785
Electrostatic energy	-212.475+/-288.985	-13.363+/-4.969	-116.754 +/- 12.300
Polar solvation energy	1493.736 +/-293.584	88.872+/-4.155	213.963 +/-31.597
Hotspot interaction binding energy	-128.661+/-87.493	-112.925+/-10.849	-63.998 +/-30.853

Table 5 was divided into parts a and b. It is important that tables are numbered in ascending numerical order: 1, 2, and 3. Since such table cannot be separated because of a main caption, it was merged and referred as Table 5. Please check if the modified presentation is appropriate. Otherwise kindly advise us on how to proceed.

## Discussion

The β-propeller domain sits atop integrin αIIbβ3 and is essential for biogenesis, maturation, and ligand binding. N15-linked glycosylation in the β-propeller represents a key quality control step in the biogenesis of αIIbβ3. Mutations in this domain have also been reported to disrupt normal intracellular trafficking by retaining mutant pro-αIIbβ3 complexes in the ER. Ligand binding of αIIbβ3 is mediated by the upper face of the “cap” region owing to the presence of Ca^2+^ and Mg^2+^ ions. In the current study, a total of 177 mutations were retrieved from the databases, such as HGMD, UniProt, ClinVar, and dbSNP, among which 60 were predicted to be deleterious using tools, such as PredictSNP, MAPP, PhD-SNP, Polyphen-1, Polyphen-2, SIFT, and SNAP^17^. Based on phenotypic severity, stability changes, and evolutionary conservation status, five mutations, namely G296R, F320S, G321W, E355K, and G401C were studied further by molecular docking analysis of both αIIb with β3 (protein-protein docking) and αIIbβ3 complex with fibrinogen (protein-peptide docking). Based on their HADDOCK scores, the E355K structure was found to have the lowest binding affinity for fibrinogen followed by the G401C structure indicating that these two mutations might affect αIIbβ3’s ligand binding function, which prompted subsequent MD simulations.

The E355K mutation had been maternally inherited by a Swiss patient with GT type 1 (severe phenotype) who was compound heterozygous with a I565T mutation in the αIIb thigh domain which had led to lower αIIbβ3 surface expression^21,22^. On the other hand, the G401C mutation had been previously reported in a French patient with GT type 2 (moderate phenotype) who was also compound heterozygous with a V934F mutation in the αIIb calf-2 domain^23^. Trajectory analysis which includes several parameters, such as RMSD, RMSF, Rg, SASA, SSE, and MMPBSA was carried out for fibrinogen-bound wild-type, E355K, and G401C complexes. RMSD and RMSF showed that the E355K structure showed higher deviations and fluctuations in comparison to both the wild-type and G401C structures. Additionally, fluctuations were observed in the C-terminal residues corresponding to the αIIb calf-2 domain. The average Rg values of the fibrinogen-bound wild-type, E355K, and G401C complexes were 3.83, 4.31, and 4.11 nm, respectively indicating that in contrast to the wild-type structure, both E355K and G401C structures had more deviations thereby compromising their structural stability. Interestingly, the E355K structure had a higher SASA value with an enlarged surface area showing that the structure is not protected from water molecules. Both fibrinogen-bound E355K and G401C complexes displayed a reduced number of hydrogen bonds in comparison to the wild-type. Of note, the G401C mutation had been previously reported to affect the Ca^2+^-binding loop by disrupting hydrogen bonds with neighboring residues^23^. Moreover, the average number of secondary structures in both were slightly higher in comparison to the wild-type, particularly β-sheets and α-helices, which could be ascribed to the large size of lysine and cysteine as compared to the respective wild-type residues. As mentioned earlier, the average binding energies for the E355K and G401C structures were found to be lower when compared to the wild-type structure. However, the hotspot interaction binding energies for these mutant structures were found to be higher than that of the wild-type which explains their absent or reduced fibrinogen binding, respectively^21^. Glutamic acid at the 355th amino acid position which is buried within the β-propeller core is critical for maintaining the domain’s structural stability. Hence, substitution with an oppositely charged lysine seemed to have perturbed stability leading to a reduced affinity to bind with fibrinogen. Whereas cysteine substitution of glycine at the 401st position did not bring about substantial changes to the interaction of surrounding residues with fibrinogen binding sites.

In conclusion, our study documents all of the mutations that have been reported so far in the αIIb β-propeller domain. While the E355K mutation has reportedly occurred in a mutation hotspot, the G401C mutation has been reported to disrupt the Ca^2+^-binding loop. MD simulations revealed that the E355K mutation strongly inhibited complex formation and fibrinogen binding with the G401C mutation having a comparably lesser impact, which could have led to the severe and moderate disease phenotypes in the Swiss and French patients, respectively. Our comprehensive in silico analysis clearly reiterates that mutations in the β-propeller domain may not only be responsible for structural changes in this domain but can also impact the overall structure and function of integrin αIIbβ3. It also provides novel insights into the dynamics of fibrinogen binding with αIIbβ3, which could pave the way for potential diagnostic and targeted therapy for Glanzmann thrombasthenia in the future.

## Methods

### Retrieval of mutations from databases

Mutations in the ITGA2B gene encoding the αIIb subunit were collected from the databases, like HGMD (www.hgmd.cf.ac.uk)^24^, Uniprot (www.uniprot.org)^25^, ClinVar (https://www.ncbi.nlm.nih.gov/clinvar/)^26^, and dbSNP (https://www.ncbi.nlm.nih.gov/snp/)^21^. The crystal structure of αIIbβ3 was retrieved from the Protein Data Bank (PDB), which has a resolution of 2.55 Å (PDB ID: 3FCS)^1^. The region of interest, namely the β-propeller domain (1-451 residues) was obtained from the αIIb subunit. Human Genome Variation Society (HGVS) nomenclature was followed throughout the study. Furthermore, these mutations were filtered based on various in silico prediction tools.

### Pathogenicity and stability analysis

Based on their deleterious nature and their impact on structural stability, the mutations were analyzed and filtered with the help of different tools, such as iStable_SEQ^18^, I-mutant_SEQ, MUpro, and CUPSAT using deltaG values and PredictSNP^17^ which is an amalgamation of several tools, like PredictSNP, SIFT, Polyphen-1, Polyphen-2, PhD-SNP, SNAP, PANTHER, MAPP, and nsSNPAnalyzer. All these tools were integrated into a consensus classifier to improve their efficiency.

### Evolutionary conservation analysis

The conservation of amino acids from an evolutionary standpoint helps correlate the tendency of amino acid(s) to be mutated and the importance of retaining amino acids for functional integrity and structural stability. The ConSurf web server was used for the conservation analysis of amino acid residues bearing mutations at specific sites^27^.

### Protein and ligand preparation

The structure of integrin αIIbβ3 ectodomain determined by X-ray crystallography (PDB: 3FCS) is composed of two globular domains, wherein αIIb and β3 are separated by a hinge region. Before proceeding with molecular docking, water and other small irrelevant molecules were removed from the structure. The protein receptor was further prepared with the addition of polar compounds and removal of partial charges. The mutations G296R, F320S, G321W, E355K, and G401C were introduced at specific locations in the PyMOL software using in silico site-directed mutagenesis (www.pymol.org*).* The fibrinogen structure was extracted from the PubChem database (CID: 90540)^28^. The ligand binds to the globular domain and the hinge region, which triggers conformational changes on the receptor to regulate its function.

### Molecular docking analysis

Molecular docking was performed for select mutations to understand the consequences due to single amino acid substitutions using the HADDOCK software which can determine interactions between protein-protein complexes. HADDOCK allows conformational changes of the molecules during complex formation, for both side chains and the backbone^29^. Initially, wild-type and mutant structures of αIIbβ3 were analyzed by protein-protein docking between αIIb and β3 subunits. This helps with identification of ambiguous interaction restraints or active residues. The docking scores were calculated based on intermolecular Van der Waals and electrostatic energies. The complexes with lowest intermolecular energy scores were selected for further analysis. Similarly, protein-peptide docking was done for the wild-type and all five mutant αIIbβ3 complexes in combination with fibrinogen using protein-ligand docking for understanding their interactions with fibrinogen^29^. The protein-protein and protein-peptide complexes were analyzed using PyMOL software to visualize changes in intermolecular interactions, such as hydrogen bonds and hydrophobic interactions between the wild-type and mutant αIIbβ3 complexes.

### Molecular dynamics simulations

The wild-type and mutant αIIbβ3 structures were subjected to molecular dynamics (MD) simulations using GROMOS96 software with 54a7 applied forcefield^30,31^. The structures of the wild-type and mutants were solvated using Simple Point Charge (SPC) in a cubic box with 1 nm away from its edge. Sodium ions were added for neutralization of the system. Energy minimization of 5000 steps were performed to correct asymmetric geometries by the steepest descent method^32^. Temperature and pressure were fixed using the Berendsen algorithm with 50,000 ps and 1000 KJ/mol/nm (T = 300 K and *P* = 1bar)^33^. The Parrinello-Rahman method was used to normalize temperature and pressure. SETTLE and LINCS algorithms were used to retain the geometry of water molecules and non-water bonds^19,20^. The wild-type and mutant structures were run for a total simulation time of about 500 ns. MD trajectories, such as root mean square deviation (RMSD), root mean square fluctuation (RMSF), solvent accessible surface area (SASA), radius of gyration (Rg), hydrogen bonds, principal component analysis (PCA), and secondary structure elements (SSE) were all performed using gmx rms, gmx rmsf, gmx sasa, gmx gyrate, gmx hbond and gmx do_dssp. The graphs were plotted using QtGrace software.

## Supplementary Information

Supplementary Material 1.

## Data Availability

The datasets generated and/or analyzed during the study have been deposited in BioStudies which can be accessed via the link, 10.6019/S-BSST1425.
