# Supplementary material for: Molecular dynamics simulations involving different β-propeller mutations reported in Swiss and French patients correlate with their disease phenotypes
Source: Sci Rep. 2024 Oct 15;14:24133. doi: 10.1038/s41598-024-75070-4 (PMC11480402; doi:10.1038/s41598-024-75070-4)
Supplement: Supplementary file 1 — Supplementary Material 1. [file 41598_2024_75070_MOESM1_ESM.docx]

**Supplementary Data**

**Table S1: List of β-propeller mutations retrieved from the various databases.**

| **S. No.** | **Accession Id** | **Source** | **Mutation** | **PredictSNP** | **MAPP** | **PhD-SNP** | **PolyPhen-1** | **PolyPhen-2** | **SIFT** | **SNAP** |
| --- | --- | --- | --- | --- | --- | --- | --- | --- | --- | --- |
| 1 | rs2048679968 | dbSNP | R3G | N | N | N | N | D | D | D |
| 2 | rs537367984 | dbSNP | A4S | N | N | N | N | D | D | N |
| 3 | CM087463 | HGMD | A4T | N | N | N | D | D | N | N |
| 4 | rs2048679771 | dbSNP | P7T | N | N | N | N | N | N | N |
| 5 | rs749730990 | dbSNP | A10V | N | N | N | N | N | D | N |
| 6 | VCV000892353 | Clinvar | E15K | N | D | N | N | D | N | D |
| 7 | rs2048679372 | dbSNP | L20P | D | - | D | D | D | D | D |
| 8 | VCV000996172 | Clinvar | P23H | D | - | N | D | D | D | D |
| 9 | rs2048679004 | dbSNP | W30S | N | N | N | D | N | N | N |
| 10 | rs1131692013 | dbSNP | W30G | N | N | D | D | N | N | D |
| 11 | CM110579 | HGMD | N33D | D | D | D | D | D | D | D |
| 12 | rs2048678755 | dbSNP | L34Q | D | D | D | D | D | D | D |
| 13 | [rs549088938](https://www.ncbi.nlm.nih.gov/snp/rs549088938) | dbSNP | D35G | D | D | D | D | D | D | D |
| 14 | rs2048678620 | dbSNP | Q38R | N | D | N | N | N | N | N |
| 15 | VCV001304284 | Clinvar | T40I | N | N | N | N | D | D | D |
| 16 | [rs5915](https://www.ncbi.nlm.nih.gov/snp/rs5915) | dbSNP | T40A | N | N | N | N | N | N | N |
| 17 | rs2048678390 | dbSNP | F41S | N | N | N | N | N | D | D |
| 18 | rs2048678361 | dbSNP | F41L | N | N | N | N | N | N | N |
| 19 | rs559994522 | dbSNP | A43V | N | N | N | N | N | D | N |
| 20 | CM136503 | HGMD | G44V | D | D | D | D | D | D | D |
| 21 | [rs376817155](https://www.ncbi.nlm.nih.gov/snp/rs376817155) | dbSNP | G47D | N | N | D | N | N | N | D |
| 22 | rs2048677906 | dbSNP | S53L | D | D | D | D | D | D | D |
| 23 | rs2048677834 | dbSNP | H57Q | D | N | D | D | D | D | D |
| 24 | VCV001302709 | Clinvar | G62E | N | N | N | N | D | N | D |
| 25 | rs2048647315 | dbSNP | I66T | D | D | N | D | D | D | D |
| 26 | VCV000953003 | Clinvar | V68M | D | D | D | D | D | D | D |
| 27 | [rs2048647129](https://www.ncbi.nlm.nih.gov/snp/rs2048647129) | dbSNP | P71R | D | D | D | D | D | D | D |
| 28 | CM125428 | HGMD | P76A | N | N | N | N | N | N | N |
| 29 | CM125429 | HGMD | S77N | N | N | N | N | N | N | N |
| 30 | VCV000323571 | Clinvar | G83D | D | D | D | D | D | N | N |
| 31 | rs2048646352 | dbSNP | L86A | N | D | N | N | D | N | N |
| 32 | CM022027 | HGMD | L86P | D | D | D | D | D | N | N |
| 33 | [rs1052533574](https://www.ncbi.nlm.nih.gov/snp/rs1052533574) | dbSNP | P88A | D | D | N | D | D | D | D |
| 34 | [rs2048646178](https://www.ncbi.nlm.nih.gov/snp/rs2048646178) | dbSNP | R90K | N | N | N | N | N | N | N |
| 35 | rs751857224 | dbSNP | Q95R | N | N | N | N | N | N | N |
| 36 | rs201860267 | dbSNP | P97H | N | N | N | N | N | N | N |
| 37 | rs2048645751 | dbSNP | V110L | N | - | N | N | N | N | N |
| 38 | rs2048641442 | dbSNP | G111S | N | - | N | N | N | N | N |
| 39 | rs2048644469 | dbSNP | T114I | N | N | N | N | N | N | N |
| 40 | rs2048644314 | dbSNP | K119N | D | N | D | D | D | D | D |
| 41 | rs2048644153 | dbSNP | A126E | D | D | D | D | D | D | D |
| 42 | rs2048643896 | dbSNP | S127A | N | D | N | N | D | D | N |
| 43 | rs372626913 | dbSNP | S127L | D | D | D | D | D | D | N |
| 44 | rs2048643792 | dbSNP | V134I | N | N | N | N | N | N | N |
| 45 | rs370013826 | dbSNP | A137G | D | D | D | D | D | D | D |
| 46 | rs750825065 | dbSNP | A139V | D | D | D | D | D | D | D |
| 47 | CM021105 | HGMD | W141G | D | D | D | D | D | D | D |
| 48 | CM153661 | HGMD | W141C | D | D | N | D | D | D | D |
| 49 | rs559891307 | dbSNP | H143P | D | D | D | D | D | D | D |
| 50 | rs200006464 | dbSNP | N145K | N | N | N | D | D | D | N |
| 51 | VCV000996207 | dbSNP | L147V | N | N | N | N | N | N | N |
| 52 | CM073147 | HGMD | K149N | N | N | N | N | N | N | N |
| 53 | VCV000634433 | Clinvar | A153T | N | - | N | N | D | D | N |
| 54 | HM060006 | HGMD | P157H | D | D | N | D | D | D | D |
| 55 | CM054765 | HGMD | G159V | D | D | D | D | D | D | D |
| 56 | rs2048641442 | dbSNP | G159S | D | D | D | D | D | D | N |
| 57 | CM119985 | HGMD | S160R | N | N | N | N | N | D | N |
| 58 | CM014712 | HGMD | C161W | D | D | D | D | D | D | D |
| 59 | rs141497408 | dbSNP | L163S | D | N | D | D | D | D | D |
| 60 | [rs2048641225](https://www.ncbi.nlm.nih.gov/snp/rs2048641225) | dbSNP | L163M | N | N | N | D | D | D | N |
| 61 | rs2048640951 | dbSNP | Q165H | N | N | N | N | N | N | N |
| 62 | rs749432805 | dbSNP | S168R | N | D | N | N | N | N | D |
| 63 | CM030893 | HGMD | R171C | D | N | D | D | D | N | D |
| 64 | CM000021 | HGMD | Y174H | D | N | D | D | D | D | D |
| 65 | CM153677 | HGMD | P176H | D | N | D | D | D | D | D |
| 66 | CM000020 | HGMD | P176A | D | N | D | D | D | D | D |
| 67 | rs368659500 | dbSNP | P176L | D | D | D | D | D | D | D |
| 68 | rs373979894 | dbSNP | T181I | N | N | N | N | N | N | N |
| 69 | rs752291386 | dbSNP | L182Q | N | N | D | D | N | N | N |
| 70 | CM156763 | HGMD | R184L | N | N | D | N | N | N | N |
| 71 | [rs2048639950](https://www.ncbi.nlm.nih.gov/snp/rs2048639950) | dbSNP | I185M | N | - | N | D | N | N | N |
| 72 | VCV000891157 | Clinvar | I185T | N | - | N | N | N | N | N |
| 73 | rs2048639566 | dbSNP | V187A | N | - | N | N | N | N | N |
| 74 | VCV001339143 | Clinvar | F191V | N | - | D | D | N | N | N |
| 75 | rs2048635909 | dbSNP | Y197C | D | N | D | D | D | D | N |
| 76 | CM153670 | HGMD | Y197N | D | N | D | D | D | D | D |
| 77 | CM043505 | HGMD | G201S | D | N | D | D | D | D | D |
| 78 | CM1712443 | HGMD | F202C | D | N | D | D | D | D | N |
| 79 | rs2048635534 | dbSNP | V206F | N | N | N | N | N | N | N |
| 80 | CM952099 | HGMD | V206D | D | D | N | D | D | D | N |
| 81 | VCV000323566 | Clinvar | T207I | D | N | D | D | D | D | D |
| 82 | rs2048634029 | dbSNP | G210R | D | N | D | D | D | D | D |
| 83 | CM981071 | HGMD | E211D | N | N | N | N | N | N | N |
| 84 | VCV001210203 | Clinvar | L214R | D | D | D | D | D | D | D |
| 85 | CM981071 | HGMD | L214P | D | D | D | D | D | D | D |
| 86 | rs267604905 | dbSNP | A216V | D | D | D | D | D | D | D |
| 87 | rs2048633615 | dbSNP | P217S | D | N | D | D | D | D | D |
| 88 | VCV001330345 | Clinvar | G219S | N | N | N | N | D | N | N |
| 89 | rs2048633560 | dbSNP | Y220H | D | N | D | D | D | D | D |
| 90 | CM981071 | HGMD | Y220C | D | N | D | D | D | D | N |
| 91 | rs2048633474 | dbSNP | F222L | D | D | D | N | N | D | N |
| 92 | rs149574963 | Unknown | R239L | N | - | D | N | N | N | N |
| 93 | rs748696622 | dbSNP | G241S | N | - | N | N | N | N | N |
| 94 | rs745448382 | dbSNP | L243F | N | N | N | N | N | N | N |
| 95 | rs2048631808 | dbSNP | W245R | N | N | N | N | N | N | N |
| 96 | rs2048631347 | dbSNP | F254L | N | D | D | N | N | N | N |
| 97 | rs2048631169 | dbSNP | Y261H | N | N | N | N | N | D | N |
| 98 | rs752656042 | dbSNP | F262S | N | N | N | N | D | N | N |
| 99 | rs368697593 | dbSNP | G264A | N | D | D | N | N | N | N |
| 100 | rs2048627164 | dbSNP | Y265H | D | N | D | D | D | D | D |
| 101 | CM021106 | HGMD | G267A | D | D | D | D | D | D | D |
| 102 | VCV001330344 | dbSNP | G267E | D | D | D | D | D | D | N |
| 103 | rs750670084 | dbSNP | A271G | N | N | D | N | D | D | N |
| 104 | CM940989 | HGMD | V272M | D | N | D | D | D | D | N |
| 105 | rs137852907 | dbSNP | G273D | D | D | D | D | D | D | D |
| 106 | rs1316600993 | dbSNP | E274G | D | N | D | D | D | D | D |
| 107 | CM156766 | HGMD | G277R | D | N | D | D | D | D | D |
| 108 | CM153669 | HGMD | T281I | N | N | N | N | N | N | N |
| 109 | rs571550200 | dbSNP | V286D | D | D | D | D | D | D | D |
| 110 | CM093511 | HGMD | G287S | D | N | D | D | D | D | N |
| 111 | rs370889770 | dbSNP | G287R | D | D | D | D | D | D | N |
| 112 | rs1057524833 | dbSNP | P289S | D | D | D | D | D | D | N |
| 113 | rs751729298 | dbSNP | T290S | N | D | D | N | N | D | N |
| 114 | CM061068 | HGMD | G296R | D | D | D | D | D | D | D |
| 115 | VCV000953005 | Clinvar | A297P | D | D | D | D | N | N | N |
| 116 | rs560777742 | dbSNP | D302N | N | N | N | N | N | N | N |
| 117 | rs1126554 | dbSNP | G313E | D | D | D | D | D | D | D |
| 118 | rs187264223 | dbSNP | A317V | D | D | D | D | D | D | D |
| 119 | rs752213788 | dbSNP | A317T | D | D | D | N | D | D | N |
| 120 | CM981072 | HGMD | S318L | D | D | D | D | D | D | D |
| 121 | G321V | HGMD | F320S | D | D | D | D | D | D | D |
| 122 | rs2048619898 | dbSNP | G321V | D | D | D | D | D | D | D |
| 123 | CM030472 | HGMD | G321W | D | D | D | D | D | D | D |
| 124 | VCV000890600 | Clinvar | H322Y | N | N | N | N | D | N | N |
| 125 | CM030472 | HGMD | V329I | N | N | N | N | D | N | N |
| 126 | VCV000996200 | Clinvar | A341V | D | D | D | D | D | D | D |
| 127 | VCV000996201 | Clinvar | A341T | D | D | D | D | D | D | D |
| 128 | CM098118 | HGMD | L343R | D | D | D | D | D | D | D |
| 129 | rs1476448476 | dbSNP | L343P | D | D | D | D | D | D | N |
| 130 | VCV000417956 | Clinvar | R348Q | N | - | N | N | N | N | N |
| 131 | VCV001338098 | Clinvar | R351Q | N | N | D | N | D | N | D |
| 132 | VCV000393125 | Clinvar | A354D | D | N | D | D | D | D | N |
| 133 | CM981073 | Uniprot | E355K | D | D | D | D | D | D | D |
| 134 | CM981073 | HGMD | E355L | D | D | D | D | D | D | D |
| 135 | VCV000426669 | Clinvar | R358S | D | D | D | D | D | D | D |
| 136 | CM950702 | HGMD | R358H | D | D | D | D | D | D | D |
| 137 | rs1026539797 | dbSNP | R358C | D | D | D | D | D | D | D |
| 138 | rs553981786 | dbSNP | P365L | N | N | N | N | N | N | N |
| 139 | rs201917696 | dbSNP | P368L | N | N | N | N | N | N | N |
| 140 | rs571678984 | dbSNP | A370T | N | - | N | N | N | N | N |
| 141 | CM021108 | HGMD | G380C | D | D | D | D | D | D | D |
| 142 | CM061067 | HGMD | G380D | D | D | D | D | D | D | D |
| 143 | VCV001030779 | Clinvar | T381I | N | N | N | D | D | N | N |
| 144 | VCV000323558 | Clinvar | Y384C | D | N | D | D | D | D | D |
| 145 | rs774609204 | dbSNP | R386Q | D | N | D | D | D | D | N |
| 146 | CM054766 | HGMD | G388S | D | D | D | D | D | D | D |
| 147 | CM093509 | HGMD | S389F | D | N | D | D | D | D | D |
| 148 | VCV000861327 | Clinvar | D396N | D | D | D | D | D | D | N |
| 149 | rs146983962 | dbSNP | D398Y | D | D | D | D | D | D | D |
| 150 | CM153663 | HGMD | R399Q | N | N | N | N | N | N | N |
| 151 | rs2048613637 | dbSNP | G401C | D | D | D | D | D | D | D |
| 152 | CM021109 | HGMD | Y402H | D | D | D | D | D | D | D |
| 153 | VCV000888906 | Clinvar | I405T | D | D | D | D | D | D | D |
| 154 | rs201156726 | dbSNP | A406V | D | D | D | D | D | D | D |
| 155 | VCV000996212 | Clinvar | A409G | D | D | D | D | D | D | D |
| 156 | rs373586333 | dbSNP | P410R | D | D | D | D | D | D | D |
| 157 | CM014714 | HGMD | Y411C | D | N | D | D | D | D | N |
| 158 | CM123722 | HGMD | G412R | D | N | D | D | D | D | D |
| 159 | rs137852909 | dbSNP | R417W | D | N | D | D | D | D | N |
| 160 | CM153665 | HGMD | G418D | D | D | D | D | D | D | D |
| 161 | rs2048592296 | dbSNP | V420E | D | D | D | D | D | D | D |
| 162 | rs532414080 | dbSNP | V420A | D | D | N | D | D | D | N |
| 163 | rs557705725 | dbSNP | R433H | N | N | N | N | N | N | N |
| 164 | rs2048591813 | dbSNP | R433C | D | N | D | D | D | D | D |
| 165 | rs2048591713 | dbSNP | V437D | D | N | N | D | D | D | D |
| 166 | rs2048591528 | dbSNP | P441L | N | D | N | N | D | N | N |
| 167 | rs2048591437 | dbSNP | G445A | N | N | N | N | N | N | N |
| 168 | CM940990 | HGMD | A447T | N | N | N | N | N | N | N |
| 169 | rs2048591163 | dbSNP | G449D | D | D | D | D | D | D | D |
| 170 | rs2048591131 | dbSNP | G454D | D | D | D | D | D | D | D |
| 171 | rs372294932 | dbSNP | A455V | N | D | N | N | D | D | N |
| 172 | rs372294932 | dbSNP | I458M | N | D | N | N | D | D | N |
| 173 | rs2048590672 | dbSNP | P464S | D | D | D | D | D | D | D |
| 174 | rs568782461 | dbSNP | V468M | D | D | D | D | D | D | D |
| 175 | rs2048587714 | dbSNP | A470G | D | D | D | D | D | D | D |
| 176 | rs375195998 | dbSNP | G472R | D | N | D | D | D | D | D |
| 177 | rs2048587484 | dbSNP | V476L | D | N | D | D | D | D | D |

D – Deleterious

N – Non-deleterious

**Table S2. Pathogenicity analysis of β-propeller mutations.**

| **S. No.** | **Mutation** | **PredictSNP** | **MAPP** | **PhD-SNP** | **PolyPhen-1** | **PolyPhen-2** | **SIFT** | **SNAP** |
| --- | --- | --- | --- | --- | --- | --- | --- | --- |
|  | N33D | D | D | D | D | D | D | D |
|  | L34Q | D | D | D | D | D | D | D |
|  | D35G | D | D | D | D | D | D | D |
|  | G44V | D | D | D | D | D | D | D |
|  | S53L | D | D | D | D | D | D | D |
|  | V68M | D | D | D | D | D | D | D |
|  | P71R | D | D | D | D | D | D | D |
|  | A126E | D | D | D | D | D | D | D |
|  | A137G | D | D | D | D | D | D | D |
|  | A139V | D | D | D | D | D | D | D |
|  | W141G | D | D | D | D | D | D | D |
|  | H143P | D | D | D | D | D | D | D |
|  | G159S | D | D | D | D | D | D | D |
|  | C161W | D | D | D | D | D | D | D |
|  | P176L | D | D | D | D | D | D | D |
|  | L214R | D | D | D | D | D | D | D |
|  | L214P | D | D | D | D | D | D | D |
|  | A216V | D | D | D | D | D | D | D |
|  | G267E | D | D | D | D | D | D | D |
|  | G273D | D | D | D | D | D | D | D |
|  | V286D | D | D | D | D | D | D | D |
|  | G296E | D | D | D | D | D | D | D |
|  | G296R | D | D | D | D | D | D | D |
|  | G313E | D | D | D | D | D | D | D |
|  | A317V | D | D | D | D | D | D | D |
|  | S318L | D | D | D | D | D | D | D |
|  | F320S | D | D | D | D | D | D | D |
|  | G321V | D | D | D | D | D | D | D |
|  | G321W | D | D | D | D | D | D | D |
|  | G321E | D | D | D | D | D | D | D |
|  | D332Y | D | D | D | D | D | D | D |
|  | D336G | D | D | D | D | D | D | D |
|  | V339A | D | D | D | D | D | D | D |
|  | A341V | D | D | D | D | D | D | D |
|  | A341T | D | D | D | D | D | D | D |
|  | L343R | D | D | D | D | D | D | D |
|  | E355K | D | D | D | D | D | D | D |
|  | E355L | D | D | D | D | D | D | D |
|  | R358S | D | D | D | D | D | D | D |
|  | R358H | D | D | D | D | D | D | D |
|  | R358C | D | D | D | D | D | D | D |
|  | G380C | D | D | D | D | D | D | D |
|  | G380V | D | D | D | D | D | D | D |
|  | G380D | D | D | D | D | D | D | D |
|  | G388A | D | D | D | D | D | D | D |
|  | G388S | D | D | D | D | D | D | D |
|  | D398Y | D | D | D | D | D | D | D |
|  | G401C | D | D | D | D | D | D | D |
|  | Y402H | D | D | D | D | D | D | D |
|  | I405T | D | D | D | D | D | D | D |
|  | A406V | D | D | D | D | D | D | D |
|  | A409G | D | D | D | D | D | D | D |
|  | P410R | D | D | D | D | D | D | D |
|  | G418D | D | D | D | D | D | D | D |
|  | V420E | D | D | D | D | D | D | D |
|  | G449D | D | D | D | D | D | D | D |
|  | G454D | D | D | D | D | D | D | D |
|  | P464S | D | D | D | D | D | D | D |
|  | V468M | D | D | D | D | D | D | D |
|  | A470G | D | D | D | D | D | D | D |

D – Deleterious

**Table S3. Stability analysis of select β-propeller mutations.**

| **S. No.** | **Accession ID** | **Mutation** | **I-Mutant** | | **MUpro** | | **I-Stable** | |
| --- | --- | --- | --- | --- | --- | --- | --- | --- |
|  |  |  | **Result** | **DDG** | **Result** | **Conf. score** | **Result** | **Conf. score** |
|  | rs2048678755 | L34Q | D | -1.71 | D | -1 | D | 0.848577 |
|  | CM136503 | G44V | D | -0.26 | D | -0.77225 | D | 0.755388 |
|  | CM125428 | P71R | D | -0.98 | D | -0.668 | D | 0.77523 |
|  | rs2048643896 | A126E | D | -0.32 | D | -0.5093 | D | 0.7377 |
|  | rs750825065 | A137G | D | -0.42 | D | -1 | D | 0.8467 |
|  | CM021105 | A139V | D | -0.17 | D | -0.2663 | D | 0.7805 |
|  | rs2048641442 | G159V | D | -0.45 | D | -0.07207 | D | 0.8132 |
|  | CM000020 | P176L | D | -0.41 | D | -0.28492 | D | 0.7762 |
|  | CM021106 | G267E | D | -0.54 | D | -0.17167 | D | 0.84338 |
|  | rs2048624107 | G296E | D | -0.67 | D | -0.11 | D | 0.8609 |
|  | CM061068 | G296R | D | -0.58 | D | -0.1761 | D | 0.838 |
|  | CM153672 | F320S | D | -2.11 | D | -0.3746 | D | 0.82 |
|  | rs2048619898 | G321V | D | -0.65 | D | -0.6238 | D | 0.7941 |
|  | CM030472 | G321W | D | -0.33 | D | -0.7686 | D | 0.7785 |
|  | rs2048619322 | D332Y | D | -0.51 | D | -0.45348 | D | 0.80351 |
|  | rs2048617102 | D336G | D | -1.51 | D | -0.2989389 | D | 0.8515 |
|  | rs2048616987 | V339A | D | -1.88 | D | -1 | D | 0.873802 |
|  | VCV000996200 | A341T | D | -0.91 | D | -1 | D | 0.740274 |
|  | rs1476448476 | L343R | D | -2.09 | D | -0.2861124 | D | 0.873903 |
|  | CM981073 | E355K | D | -1.35 | D | -1 | D | 0.833752 |
|  | CM054766 | G388S | D | -1.54 | D | -0.2922696 | D | 0.750431 |
|  | CM153663 | G401C | D | -1.35 | D | -1 | D | 0.774686 |
|  | VCV000888906 | A406V | D | -0.17 | D | -0.2954434 | D | 0.846034 |
|  | VCV000996212 | P410R | D | -1.06 | D | -0.7072345 | D | 0.738268 |
|  | rs2048590672 | P464S | D | -1.88 | D | -1 | D | 0.904869 |
|  | rs568782461 | V468M | D | -1.08 | D | -0.2048153 | D | 0.773162 |
|  | rs2048587714 | A470G | D | -1.22 | D | -1 | D | 0.754603 |

D – Deleterious / Destabilizing

**Table S4. Evolutionary conservation analysis of select β-propeller mutations.**

| **Accession number** | **Mutation** | **3FCS** | **I-Mutant** | **MUpro** | **I-Stable** | **PredictSNP** | **MAPP** | **PhD-SNP** | **PolyPhen-1** | **PolyPhen-2** | **SIFT** | **ConSurf** | **Refs.** |
| --- | --- | --- | --- | --- | --- | --- | --- | --- | --- | --- | --- | --- | --- |
| CM136503 | G44V | G13V | D | D | D | D | D | D | D | D | D | 9 | ^31^ |
| CM000020 | P176L | P145L | D | D | D | D | D | D | D | D | D | 9 | ^32^ |
| CM021106 | G267E | G236E | D | D | D | D | D | D | D | D | D | 9 | ^5^ |
| CM061068 | G296R | G265R | D | D | D | D | D | D | D | D | D | 9 | ^33^ |
| CM153672 | F320S | F289S | D | D | D | D | D | D | D | D | D | 8 | ^34^ |
| CM030472 | G321W | G290W | D | D | D | D | D | D | D | D | D | 9 | ^35^ |
| rs1476448476 | L343R | L312R | D | D | D | D | D | D | D | D | D | 8 | ^36^ |
| CM981073 | E355K | E324K | D | D | D | D | D | D | D | D | D | 9 | ^37^ |
| CM153663 | G401C | G370C | D | D | D | D | D | D | D | D | D | 9 | ^35^ |

D – Deleterious / Destabilizing


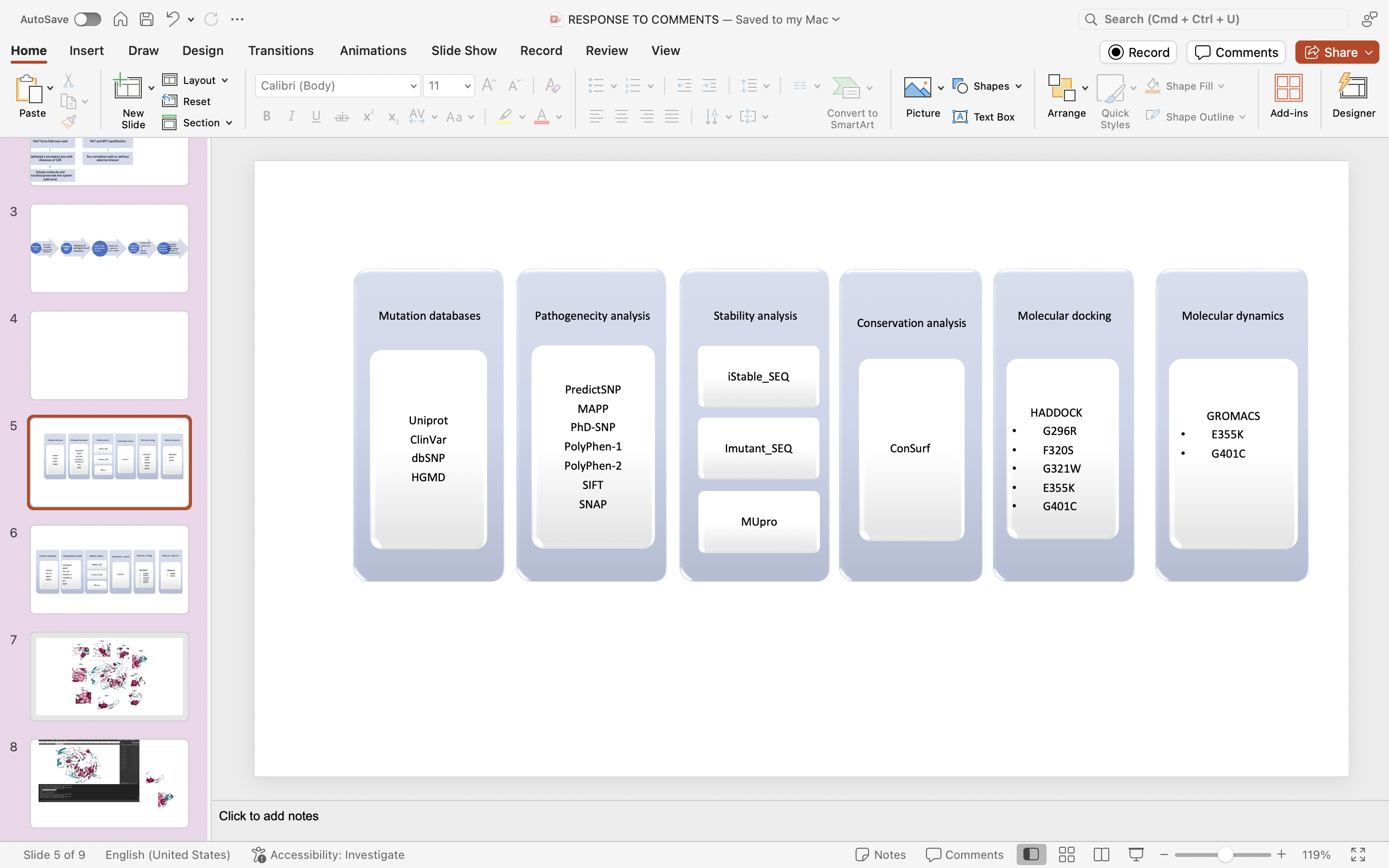


**Figure S1. Flowchart depicting the various bioinformatics tools used in the study.**
